# The complete genome sequence of the *Pediococcus siamensis* type strain MCH3-2^T^ (= JCM 13997^T^ = LMG 24279^T^) Tanasupawat et al. 2007, 1372^VP^

**DOI:** 10.1128/mra.00064-25

**Published:** 2025-08-27

**Authors:** Gyu-Sung Cho, Erik Brinks, Charles M. A. P. Franz

**Affiliations:** 1 Department of Microbiology and Biotechnology, Max Rubner-Institute, Federal Institute for Nutrition and Food 125569 , Kiel, Germany; University of Strathclyde, Glasgow, United Kingdom

**Keywords:** *Pediococcus siamensis*, hybrid assembly, complete genome

## Abstract

The complete genome sequence of *Pediococcus siamensis* MCH3-2^T^ (= JCM 13997^T^ = LMG 24279^T^) is reported. A hybrid assembly combining Illumina short-read and Nanopore long-read data using Unicycler revealed a circular chromosome and two extra-chromosomal contigs, comprising 2219 CDS, 62 tRNAs, and 12 rRNAs.

## ANNOUNCEMENT

*Pediococcus siamensis* was first described by Tanasupwat et al. (2007), and the type strain MCH3-2^T^ was isolated from fermented tea leaves (*miang*) in northern Thailand (1). Based on 16S rRNA gene phylogeny, the closest relative of *P. siamensis* MCH3-2^T^ was the *Pediococcus cellicola* Z-8^T^ type strain, followed by *Pediococcus damnosus* DSM 20331^T^ and *Pediococcus inopinatus* LMG 11409^T^ (1). To date, the genome sequence of the *P. siamensis* MCH3-2^T^ type strain has not been available for taxonomic or functional investigations. Freeze-dried *P. siamensis* LMG 24279^T^ was purchased from LMG Culture Collection (Universiteit Gent, Gent, Belgium) and routinely cultured aerobically in de Man, Rogosa and Sharpe broth (Merck, Darmstadt, Germany) at 30°C for 18 h. Genomic DNA was extracted twice from an 18 h grown culture using two different kits—the Omega Bacterial DNA Kit (VWR, Darmstadt, Germany) for Illumina short-read sequencing, and Genomic Micro AX Bacterial Gravity Kit (A&A Biotechnology, Gdynia, Poland) for Nanopore long-read sequencing. The concentration of extracted DNA was measured using a Qubit 3 (Thermo Fisher Scientific, Wesel, Germany). The sequencing library was prepared using the Illumina DNA Prep Kit (Illumina, Munich, Germany) followed by paired-end sequencing with the MiSeq Reagent Kit v2 (Illumina) on a MiSeq sequencing platform (Illumina). For long-read DNA sequencing, the DNA library was randomly prepared without shearing, according to the Native Barcoding Kit 96 v. 14 SQK-NBD114.96 (Oxford Nanopore Technologies, Oxford, UK) instructions and was sequenced on the PromethION 2 Solo platform (Oxford Nanopore Technologies). The paired-end Illumina raw sequence (2 × 151 bp) data were trimmed using Trimmomatic (v. 0.39) with default parameters (2), generating 368,499 paired-end reads (109,479,038 bp). FASTQ files were generated from the raw POD5 by basecalling and demultiplexing with Dorada software (v. 0.6.4, https://github.com/nanoporetech/dorado) with --hac parameter and filtered by chopper (v. 0.9.0; parameter: minlength 500; quality Q20) (3), generating 25,984 reads (225,116,792 bp), average 8.6 kb, *N*_50_ = 11,848. A hybrid assembly with short- and long-read data was performed using Unicycler (4) (v. 0.5.1; parameter: conservative). The predicted genome coverage depth was 147×, and three circular contigs were generated, with N_50_ of 2,249,876 bp. After *de novo* assembly, all contigs were annotated using the NCBI Prokaryotic Genome Annotation Pipeline (5) (v. 6.9). Acquired antibiotic resistance genes and plasmid replicon type were identified using the Staramr pipeline (6) (v. 0.10.0) with default parameters.

The genome sequence of *P. siamensis* LMG 24279^T^ possessed a single circular chromosome and two extrachromosomal circular contigs, with a total length of 2,298,821 bp and a G+C content of 41.07 mol%. It contained 2,231 protein-coding sequences, 62 tRNAs, 12 rRNAs, and 106 repeat regions. No acquired antibiotic resistance genes could be detected. The size of the chromosome was 2,249,876 bp with 41.04 mol% G+C content, while the sizes of the extrachromosomal contigs were 37,879 bp and 11,066 bp, and these had 43.84 and 38.71 mol% G+C contents, respectively. The small plasmid possessed a replication protein (rep28), while the large plasmid did not contain a Rep protein with over 80% similarity. Genome-based identification using the Genome to Genome Distance Calculator (GGDC) (7) (v. 3.0) and the OrthoANI (8) (v. 0.93.1) revealed similarity values with closely related *Pediococcus* species (Table 1).

**TABLE 1:** Average nucleotide identity (ANI) and *in silico* DDH (dDDH) values determined between *P. siamensis* LMG 24279^T^ and closely related type strains

	ANI (%)	dDDH (%)
*P. claussenii* DSM 14800^T^	68.9	25.0
*P. pentosaceus* ATCC 33316^T^	69.0	22.7
*P. argentinicus* CCUG 54535^T^	69.6	22.4
*P. acidilactici* CCUG 32235^T^	68.7	22.3
*P. stilesii* DSM 18001^T^	68.6	22.2
*P. ethanolidurans* DSM 22301^T^	76.4	20.4
*P. damnosus* DSM 20331^T^	75.1	20.0

## Data Availability

The whole genome sequencing project has been deposited at NCBI under the BioProject accession number PRJNA1208221 and the Biosample accession number SAMN46169686. Raw sequences have been deposited to the Sequencing Read Archive under the accession numbers SRX28417056 (Nanopore) and SRX27423969 (Illumina). The sequenced genomic data have been deposited in DDBJ/EMBL/GenBank under the accession numbers CP178375, CP178376, and CP178377.
